# Pinostrobin and Tectochrysin Conquer Multidrug-Resistant Cancer Cells via Inhibiting P-Glycoprotein ATPase

**DOI:** 10.3390/ph16020205

**Published:** 2023-01-29

**Authors:** I-Ting Wu, Chan-Yen Kuo, Ching-Hui Su, Yu-Hsuan Lan, Chin-Chuan Hung

**Affiliations:** 1 Department of Pharmacy, China Medical University, No. 100, Sec. 1, Jingmao Rd., Beitun District, Taichung 406040, Taiwan; 2 Department of Research, Taipei Tzu Chi Hospital, Buddhist Tzu Chi Medical Foundation, New Taipei City 231405, Taiwan; 3 Department of Pharmacy, China Medical University Hospital, No. 2, Yude Rd., North District, Taichung 404332, Taiwan; 4 Department of Healthcare Administration, Asia University, 500, Lioufeng Rd., Wufeng, Taichung 41354, Taiwan

**Keywords:** drug efflux, ATPase, pinostrobin, tectochrysin, Flavonoid, multidrug resistance, P-glycoprotein

## Abstract

Enhanced drug efflux through ATP-binding cassette transporters, particularly P-glycoprotein (P-gp), is a key mechanism underlying multidrug resistance (MDR). In the present study, we investigated the inhibitory effects of pinostrobin and tectochrysin on P-gp in MDR cancer cells and the underlying mechanisms. Fluorescence substrate efflux assays, multidrug resistance 1 (MDR1) shift assays, P-gp ATPase activity assays, Western blotting, and docking simulation were performed. The potential of the test compounds for MDR reversal and the associated molecular mechanisms were investigated through cell viability assay, cell cycle analysis, apoptosis assay, and further determining the combination index. Results demonstrated that pinostrobin and tectochrysin were not the substrates of P-gp, nor did they affect the expression of this transporter. Both compounds noncompetitively inhibited the efflux of rhodamine 123 and doxorubicin through P-gp. Furthermore, they resensitized MDR cancer cells to chemotherapeutic drugs, such as vincristine, paclitaxel, and docetaxel; thus, they exhibited strong MDR reversal effects. Our findings indicate that pinostrobin and tectochrysin are effective P-gp inhibitors and promising candidates for resensitizing MDR cancer cells.

## 1. Introduction

Cancer is the primary reason for death worldwide. Although various options, such as surgery, radiation therapy, immunotherapy, and hormone therapy, can be used for treating cancer, chemotherapy remains the predominant treatment option. Multidrug resistance (MDR) is a major concern in chemotherapy. Numerous mechanisms underlie the development of MDR, such as enhanced DNA repair capacity, increased drug efflux, mutated drug targets, and uncontrolled cell cycles, etc. [1,2]. Among these mechanisms, enhanced drug efflux is a key process.

In the cell membrane, ATP-binding cassette (ABC) transporters, such as P-glycoprotein (P-gp/MDR1/ABCB1), breast cancer resistance protein (BCRP; ABCG2), and multidrug resistance-associated protein 1 (MRP1; ABCC1), regulate the absorption, distribution, and excretion of various compounds. High expression levels of ABC transporters on the surface of cancer cells may reduce chemotherapeutic drug penetration and intracellular concentration. For instance, P-gp is often expressed at high levels on the surface of endothelial cells, thus hindering the penetration of chemotherapeutic drugs. P-gp and BCRP efflux various anticancer agents that are structurally and functionally unrelated, such as taxanes, vinca alkaloids, anthracyclines, colchicine, and mitoxantrone, thus reducing intracellular drug accumulation and drug efficacy. Many types of cancer express ABC transporters, such as colorectal cancer, liver cancer, acute myeloid (AML), and breast cancer. Approximately 35% of acute myeloid leukemia and 71% of relapsed breast cancer cases involve the overexpression of P-gp, leading to the failure of anticancer therapies [3,4,5]. Furthermore, chemotherapy failure may result from inflammation and tumor gene mutations [1,2].

**Figure 1:**
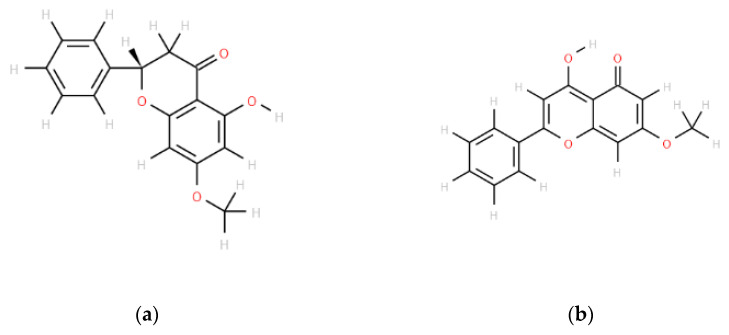
Structures of pinostrobin (**a**) and tectochrysin (**b**).

Among phytochemicals, alkaloids are the most extensively studied secondary metabolites in the context of MDR [6]. Flavonoids, another important group of secondary metabolites, exhibit in vitro anti-inflammatory, antiviral, antiallergic, anticarcinogenic, antimutagenic, and osteogenetic activities. To date, various secondary metabolites, such as genistein, naringenin, and curcumin, have been reported to affect ABC transporters [7]. Pinostrobin (Figure 1a), a dietary bioflavonoid isolated from *Pinus strobus*, exhibits antiviral (against herpes simplex virus-1) and antibacterial (against *Helicobacter pylori*) activities. This bioflavonoid also exhibits anti-inflammatory activities, including the inhibition of the expression of tumor necrosis factor (TNF)-α, interleukin-1β, cyclooxygenase (COX)I, and COXII [8]. Furthermore, pinostrobin inhibits the formation of focal and cell adhesions to reduce the migration of malignant breast epithelial cells [9]. This finding indicates that pinostrobin can be used to control the progression of advanced breast cancer. Previous studies have reported that this compound exhibits no substantial cytotoxicity in drug-sensitive and drug-resistant cell lines [8,9,10,11].

Tectochrysin (Figure 1b) is isolated from *Alpinia oxyphylla*. This compound exerts antiproliferative effects on human colorectal adenocarcinoma cells by inducing the expression of DR3, DR4, Fas, and proapoptotic proteins and inhibiting the activity of NF-κB [12]. Tectochrysin alleviates Aβ1–42-induced impairments of spatial learning and memory in mice, which indicates its therapeutic potential for Alzheimer’s disease [13]. The combination of tectochrysin with cetuximab inhibits epidermal growth factor receptor signaling to control the growth of cancer cells [14]. It is a potent inhibitor of ABCG2-mediated drug efflux [15].

The effects of pinostrobin and tectochrysin on MDR reversal and P-gp activity have been minimally explored in previous studies [15,16], but their mechanisms of action have not been investigated in depth. Therefore, in the present study, we assessed the effects of pinostrobin and tectochrysin on the expression and function of P-gp and its kinetics to elucidate the underlying molecular mechanisms. In addition, we investigated the efficacy of the combination of pinostrobin or tectochrysin with chemotherapeutic drugs in MDR cancer cells; for this, the potential of pinostrobin and tectochrysin for MDR reversal was evaluated.

## 2. Results

### 2.1. Inhibitory Effects of Pinostrobin and Tectochrysin on Efflux Function and Kinetic Mechanisms

**Figure 2:**
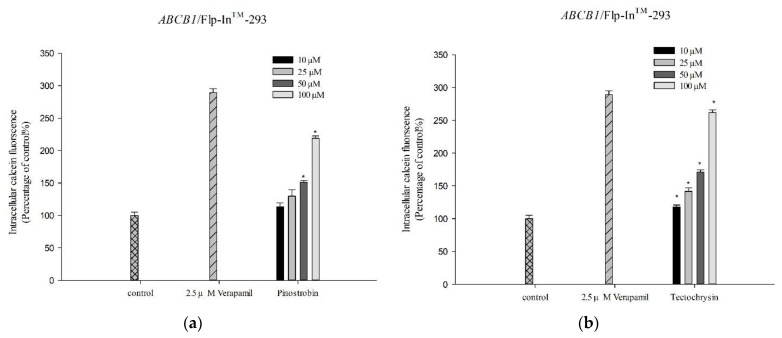
Inhibitory effects of pinostrobin (**a**) and tectochrysin (**b**) on human P-gp. Verapamil is a known P-gp inhibitor and was used as a positive control in our study. Data are presented as mean ± standard error. * indicates comparison with the control; *p* < 0.05.

To determine whether pinostrobin and tectochrysin affected the efflux function of human P-gp, we performed calcein-AM uptake assays. Calcein-AM, a nonfluorescent compound, penetrates viable cells and is hydrolyzed by intracellular esterases to produce fluorescent calcein. Calcein is a substrate of P-gp; therefore, the inhibition of P-gp interferes with calcein efflux, which, in turn, prevents the accumulation fluorescent calcein in cells. As shown in Figure 2, the intensity of intracellular calcein fluorescence markedly increased with the increasing concentrations of pinostrobin and tectochrysin. Therefore, pinostrobin and tectochrysin may inhibit the efflux function of P-gp.

**Figure 3:**
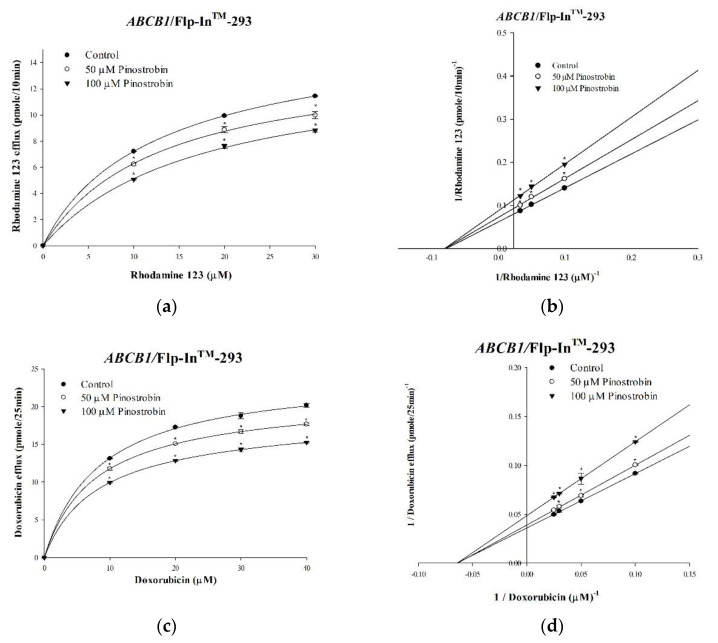
Pinostrobin−mediated inhibition: kinetic mechanisms underlying the P-gp-mediated effluxes of rhodamine 123 and doxorubicin. The saturation curve graphs indicate that the concentration-dependent effects of pinostrobin on the effluxes of rhodamine 123 (**a**) and doxorubicin (**c**) followed Michaelis–Menten kinetics. The Lineweaver−Burk plots present kinetics of the effluxes of rhodamine 123 (**b**) and doxorubicin (**d**). Data are presented as mean ± standard. * indicates comparison with the untreated control; *p* < 0.05.

**Figure 4:**
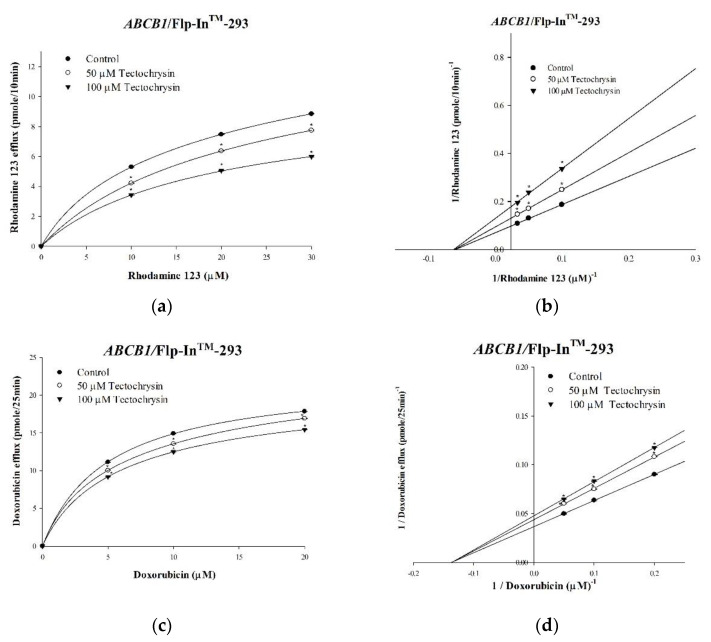
Tectochrysin−mediated inhibition: kinetic mechanisms underlying the P-gp-mediated effluxes of rhodamine 123 and doxorubicin. The saturation curve graphs indicate that the concentration-dependent effects of tectochrysin on the effluxes of rhodamine 123 (**a**) and doxorubicin efflux (**c**) followed Michaelis–Menten kinetics. The Lineweaver−Burk plots present the kinetics of the effluxes of rhodamine 123 (**b**) and doxorubicin (**d**). Data are presented as mean ± standard deviation. * indicates comparison with the untreated control; *p* < 0.05.

To investigate the enzyme kinetics associated with the effects of pinostrobin and tectochrysin on human P-gp, we performed rhodamine 123 and doxorubicin efflux assays. Rhodamine 123 and doxorubicin are well-known fluorescent substrates of P-gp. Their effluxes followed Michaelis–Menten kinetics; the kinetic parameters were analyzed. Although pinostrobin and tectochrysin decreased the maximal efflux rate (V_m_) of rhodamine 123, the compounds did not affect the affinity (K_m_) of P-gp (Table 1 and Table 2; Figure 3 and Figure 4). These findings suggested that pinostrobin and tectochrysin noncompetitively inhibited the efflux of rhodamine 123 through P-gp. Similar results were obtained for doxorubicin. Thus, pinostrobin and tectochrysin noncompetitively inhibit drug efflux through human P-gp.

### 2.2. Effects of Pinostrobin and Tectochrysin on the Conformation, Atpase Activity, and Expression of P-Gp

**Figure 5:**
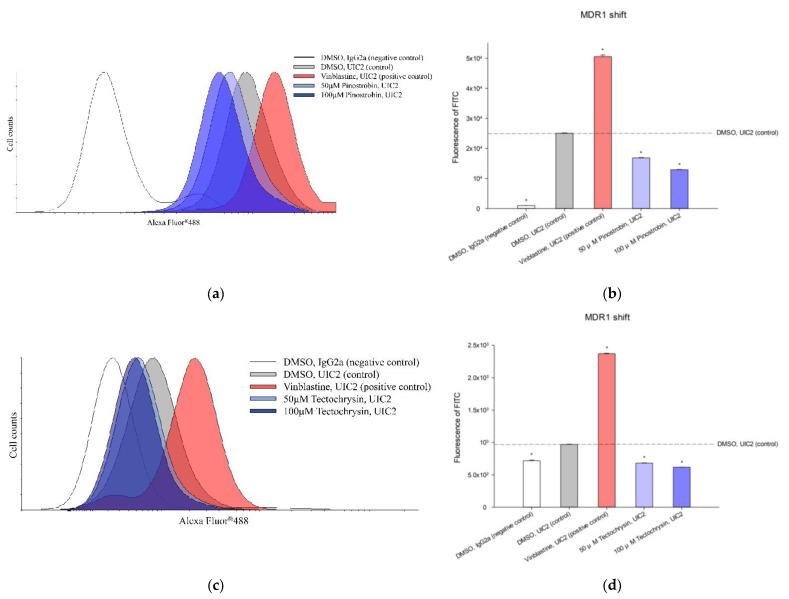
Effects of pinostrobin (**a**,**b**) and tectochrysin (**c**,**d**) on the conformation of human P-gp. The peak graphs (**a**,**c**) display the peak positions of fluorescence for different treatments, and the bar charts (**b**,**d**) present statistical data. The MDR1/ABCB1(UIC2) antibody is sensitive to the open conformation of P-gp. IgG2a antibody served as the negative control, whereas vinblastine served as the positive control. Data are presented as mean ± standard error values. * indicates comparison with the dimethyl sulfoxide control; *p* < 0.05.

Whether pinostrobin and tectochrysin are the substrates of human P-gp was evaluated through the MDR1 shift assay. Vinblastine, a substrate of human P-gp, increased the fluorescence of cells treated with MDR1/ABCB1 antibody (UIC2); thus, it was regarded as a positive control. As shown in Figure 5, although pinostrobin and tectochrysin affected the fluorescence of cells treated with UIC2, they markedly reduced, and not increased, the fluorescence compared with the control. Hence, pinostrobin or tectochrysin is not a substrate of human P-gp.

**Figure 6:**
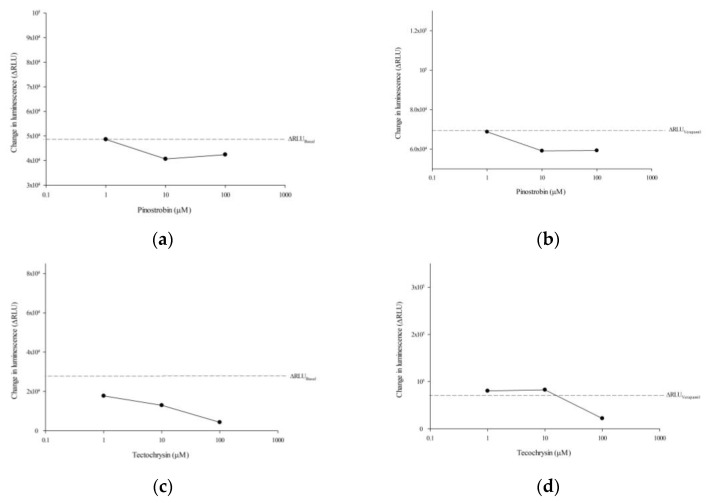
Effects of pinostrobin (**a**,**b**) and tectochrysin (**c**,**d**) on the ATPase activity of human P-gp. The left panels indicate the effects of pinostrobin (**a**) and tectochrysin (**c**) on ATPase activity, whereas the right panels indicate the effects of pinostrobin (**b**) and tectochrysin (**d**) on verapamil-induced ATPase activity. ΔRLU represents the changes in luminescence. Data are presented as mean ± standard error.

To investigate whether pinostrobin and tectochrysin affected the activity of human P-gp ATPase, we performed the Pgp-Glo^TM^ assay. As shown in Figure 6, both pinostrobin and tectochrysin inhibited the basal ATPase activity of P-gp. Furthermore, pinostrobin and high concentration of tectochrysin inhibited the verapamil-stimulated ATPase activity of P-gp (Figure 6).

**Figure 7:**
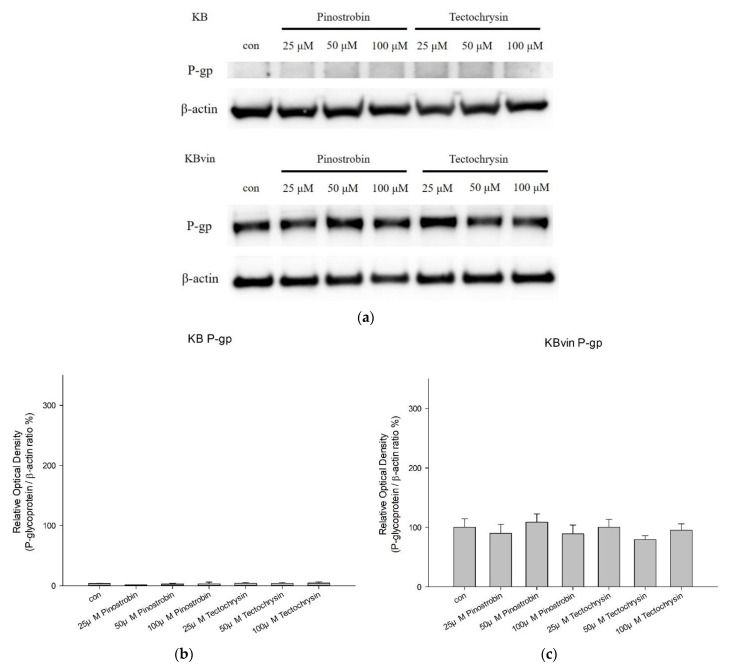
Effects of pinostrobin and tectochrysin on the expression of P-gp. (**a**) Results of the comparison between the control and test compounds (pinostrobin and tectochrysin) in terms of Western blotting data obtained using the isolates of pinostrobin or tectochrysin -treated (48 h) KB and KBvin cells. Bar charts presenting the changes in relative optical density (compared with the findings in the KBvin control group) in KB (**b**) and KBvin (**c**) cells. Data are presented as mean ± standard error.

The effects of pinostrobin and tectochrysin on the expression levels of P-gp were investigated through Western blotting. After 48 h of incubation with pinostrobin or tectochrysin, the expression level of human P-gp in human oral squamous carcinoma (KB) cells was inapparent (Figure 7). The expression levels of P-gp were slightly affected in the resistant KBvin cell line; however, no significant differences were observed between the control and treatment groups (Figure 7). Hence, pinostrobin and tectochrysin may not affect the expression levels of P-gp.

### 2.3. Potential of Pinostrobin and Tectochrysin for MDR Reversal and Underlying Mechanisms

As shown in Table 3, the IC_50_ values of pinostrobin and tectochrysin in a total of four cell lines were considerably higher than 100 μM; hence, in the subsequent experiments, we used the concentration of < 100 μM. The IC_50_ values of paclitaxel, vincristine, and docetaxel against KBvin and *ABCB1*/Flp-In^TM^-293 cells were significantly higher than those against human cervical adenocarcinoma (HeLa S3) and Flp-In^TM^-293 cells, respectively. Thus, the KBvin and *ABCB1*/Flp-In^TM^-293 cell lines were highly resistant to chemotherapeutic drugs. When the concentrations of the test compounds were reduced in the combination treatments, the corresponding IC_50_ values of the resistant cell lines reduced synergistically, and the reversal fold values increased substantially (Table 4 and Table 5). Drug–drug interactions between pinostrobin or tectochrysin and chemotherapeutic drugs were determined through combination index (CI) analysis. As shown in Table 6, the CI values of pinostrobin and three chemotherapeutic drugs ranged from 0.092 to 0.523, indicating the synergist effects of the combination treatments. The CI values of tectochrysin and the three chemotherapeutic drugs were similar (0.023–0.839; Table 7). Therefore, pinostrobin and tectochrysin may exert synergistic effects on MDR reversal.

**Figure 8:**
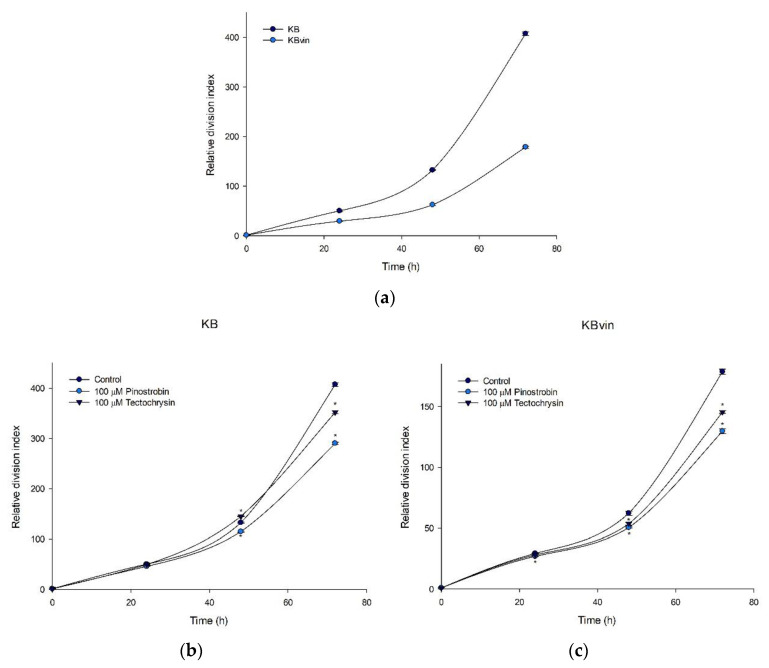
Effects of pinostrobin and tectochrysin on the proliferation of cells. The relative division index was inverse-normalized using the 0-h fluorescence level. (**a**) Basal division index of the KB and KBvin cell lines. (**b**,**c**) Curved graphs presenting the index of the control, pinostrobin, and tectochrysin treatments in the KB and KBvin cell lines, respectively. Data are presented as mean ± standard error. * indicates comparison with the control; *p* < 0.05.

The relative division index values of the KB and KBvin cell lines increased in a time-dependent manner. With time, the fluorescence peak shifted toward the left because the fluorescence due to carboxyfluorescein succinimidyl ester (CFSE) decreased with cell division (Figure 8a and Figure S1). In KB cells, although tectochrysin increased the relative division index value after 48 h, pinostrobin and tectochrysin reduced the index value after 72 h. By contrast, the test compounds substantially decreased the index values both at 48 h and 72 h in KBvin cells (Figure 8b,c). As shown in the overlapped peak graph, compared with the findings obtained through the control treatment, the peaks of cells treated with the test compounds, particularly pinostrobin, shifted toward the right. Therefore, pinostrobin and tectochrysin may inhibit the proliferation of cells. The effects of pinostrobin on cell proliferation were stronger than those of tectochrysin (Figure S1).

**Figure 9:**
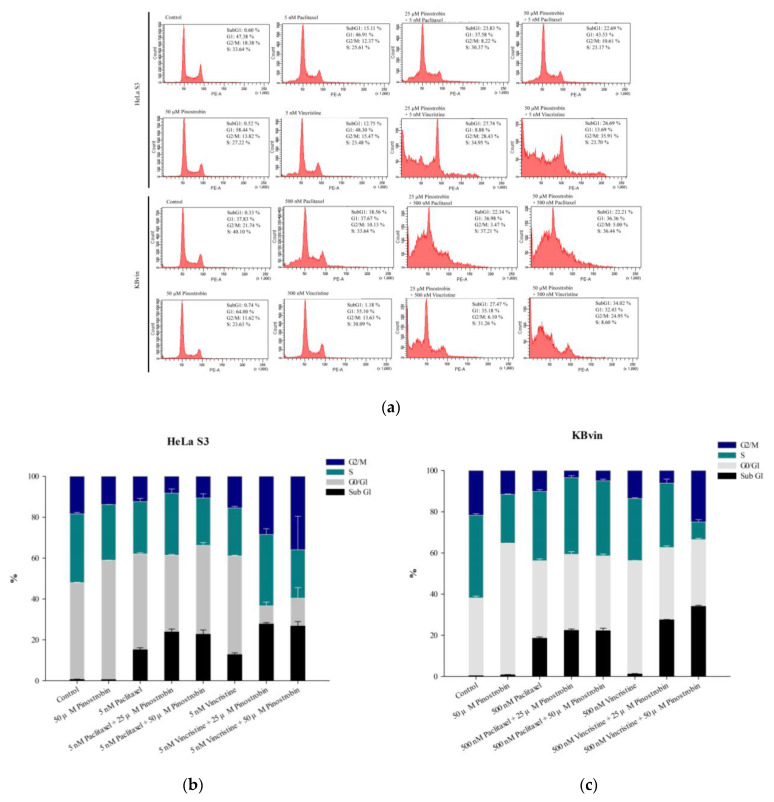
Effects of pinostrobin on the cell cycle in the HeLa S3 and KBvin cell lines. (**a**) Cell cycle was assessed through flow cytometry performed after the treatment of the aforementioned cells with pinostrobin for 48 h. The bar charts depict the proportions of HeLa S3 (**b**) and KBvin (**c**) cells in various phases of the cell cycle. Data are presented as mean ± standard error.

**Figure 10:**
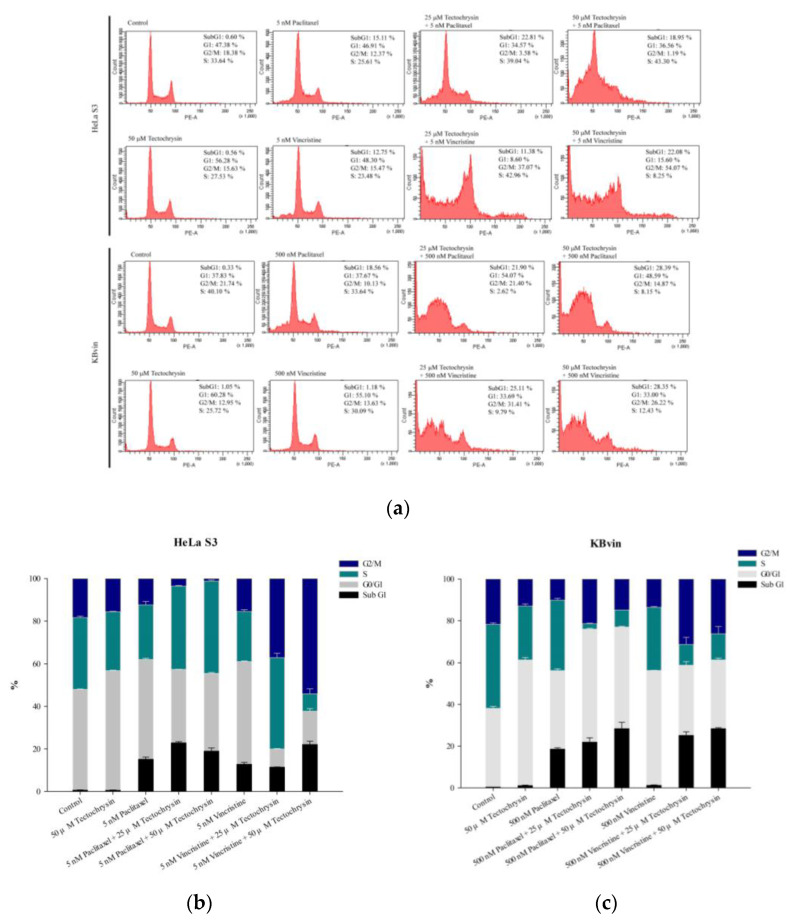
Effects of tectochrysin on the cell cycle in the HeLa S3 and KBvin cell lines. (**a**) Cell cycle was assessed through flow cytometry performed after the treatment of HeLa S3 and KBvin cells with tectochrysin for 48 h. The bar charts depict the proportions of HeLa S3 (**b**) and KBvin (**c**) cells in various phases of the cell cycle. Data are presented as mean ± standard error.

As shown in Figure 9 and Figure 10, the treatment of cells with only paclitaxel or vincristine led to normal cell cycle distribution; similar results were obtained for pinostrobin and tectochrysin treatments. The combination of pinostrobin and chemotherapeutic drugs increased the proportion of apoptotic cells and reduced the proportion of cells in the G1 phase of the cell cycle. By contrast, the combination of tectochrysin and chemotherapeutic drugs increased the proportions of HeLa S3 cells in the S phase of the cell cycle and those of KBvin cells in the sub-G1 phase of the cell cycle.

**Figure 11:**
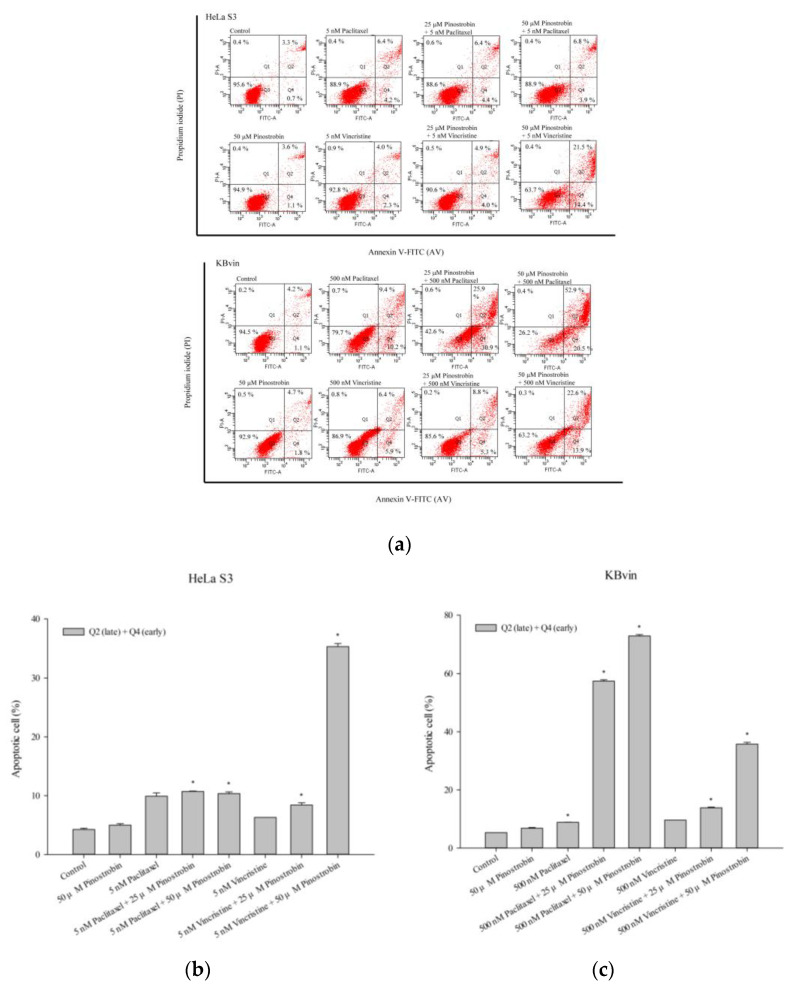
Death pattern of HeLa S3 and KBvin cells. (**a**) Apoptotic cells were assessed through flow cytometry performed after the treatment of HeLa S3 and KBvin cells with pinostrobin for 48 h. The bar charts depict the proportions of apoptotic cells in the HeLa S3 (**b**) and KBvin (**c**) cell lines. Data are presented as mean ± standard. * indicates comparison with the control; *p* < 0.05.

**Figure 12:**
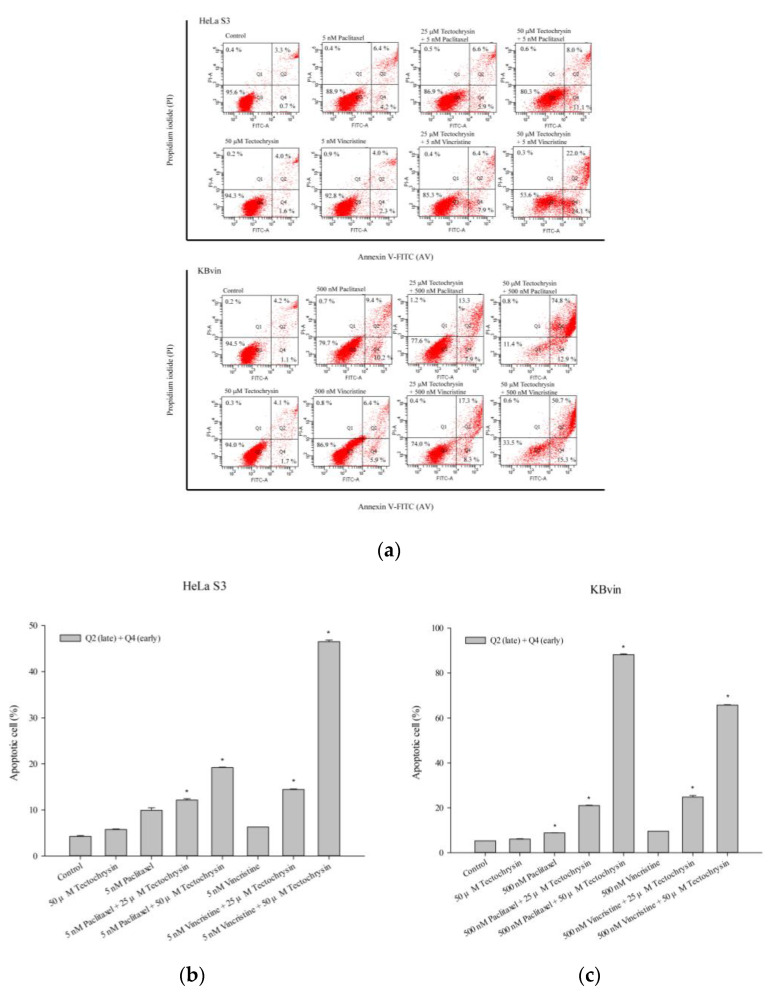
Death pattern of HeLa S3 and KBvin cells. (**a**) Apoptotic cells were assessed through flow cytometry performed after the treatment of the HeLa S3 and KBvin cells with tectochrysin for 48 h. The bar charts depict the proportions of apoptotic cells in the HeLa S3 (**b**) and KBvin (**c**) cell lines. Data are presented as mean ± standard deviation. * indicates comparison with the control; *p* < 0.05.

The pattern of cell death was assessed using the HeLa S3 and KBvin cancer cell lines. As shown in Figure 11 and Figure 12, the combination treatments induced early apoptosis and late apoptosis in the two cell lines. In MDR KBvin cells, the proportion of live cells decreased, and that of late apoptotic cells markedly increased. Hence, pinostrobin and tectochrysin may enhance the cytotoxic effects of chemotherapeutic drugs.

### 2.4. Molecular Docking of Pinostrobin and Tectochrysin on P-gp

**Figure 13:**
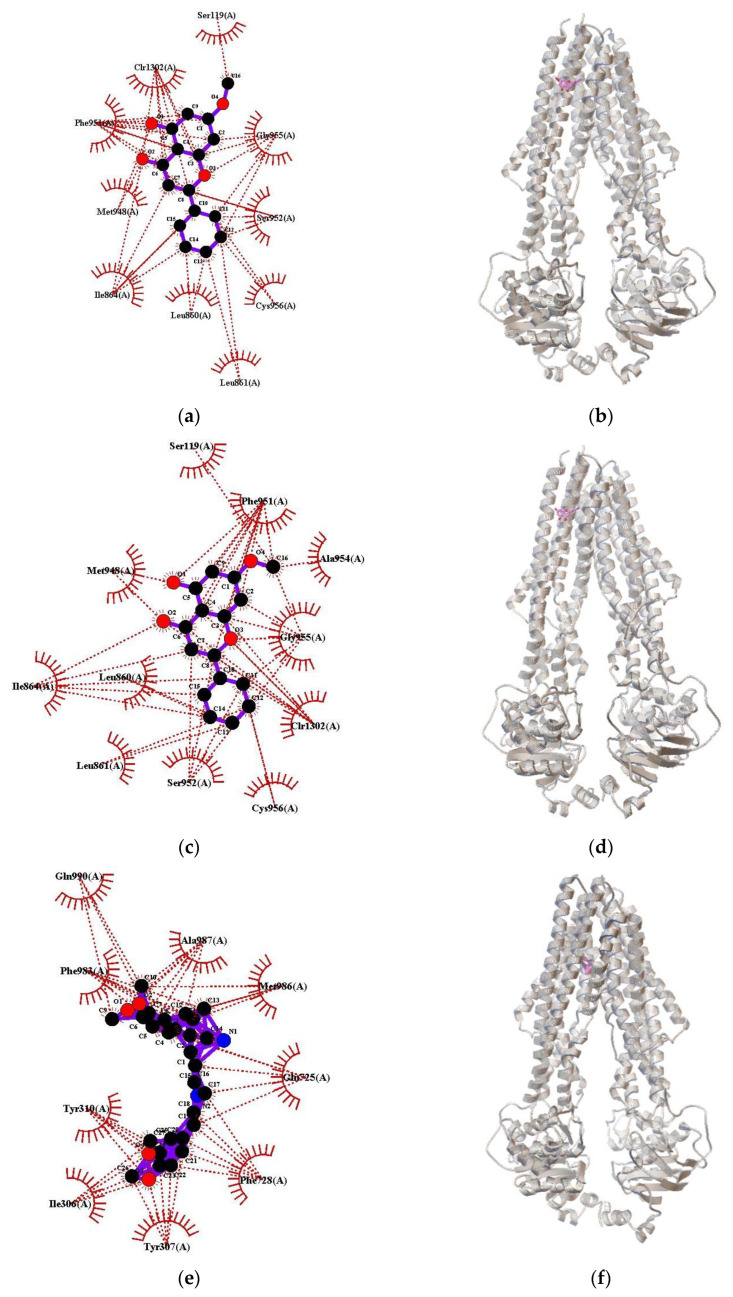
Two-dimensional and three-dimensional models of molecular docking of pinostrobin, tectochrysin, and verapamil on P-gp. Two-dimensional models showing the interactions among the ligands pinostrobin (**a**), tectochrysin (**c**), and verapamil (**e**). Three-dimensional models showing the adducts of pinostrobin (**b**), tectochrysin (**d**), and verapamil (**f**) with P-gp. Verapamil is a P-gp inhibitor.

The best docking interactions between the test compounds and human P-gp were simulated using AutoDock 4.2.6. Table 8 and Figure 13 present the binding energy levels and amino acids involved in the docking interactions and hydrogen bonding. Pinostrobin, tectochrysin, and verapamil did not form hydrogen bonds with the amino acids in P-gp. For pinostrobin and tectochrysin, similar amino acids were involved in the interactions. Moreover, the value of the lowest binding energy was higher for verapamil, a standard inhibitor of P-gp, than for the test compounds. Therefore, the affinities of pinostrobin and tectochrysin toward human P-gp may be better than that of verapamil.

## 3. Discussion

In the present study, pinostrobin and tectochrysin exerted inhibitory effects on P-gp. The results of the P-gp ATPase activity assay and kinetic analysis revealed that pinostrobin and tectochrysin inhibited drug efflux through P-gp by hindering its ATPase activity and noncompetitively inhibiting the transport of drugs, namely rhodamine 123 and doxorubicin. In addition, pinostrobin and tectochrysin restored the sensitivity of MDR cancer cells to chemotherapeutic drugs, such as paclitaxel, vincristine, docetaxel, thus exhibiting strong MDR reversal activity. These findings suggest that pinostrobin and tectochrysin are promising candidates for treating cancer synergistically.

In chemotherapy, the development of MDR remains the predominant obstacle. Enhanced drug efflux by ABC transporters is a key mechanism underlying MDR. The induction of P-gp expression in cancer cells is associated with poor clinical outcomes and reduced chemotherapeutic responses in various cancer types. Thus, P-gp has been regarded as a potential target for overcoming MDR in cancer [17]. Although early inhibitors, such as valspodar, were shown to inhibit cytochrome P450 and lead to chemotherapy dose reductions, third generation inhibitors were nontoxic and effective. However, the clinical benefits of P-gp inhibitors are yet to be confirmed owing to the absence of P-gp in patients’ tumors. These clinical trials did not examine whether these tumors expressed P-gp or not. Tariquidar could reverse the doxorubicin-resistance in mouse model [17,18,19,20].

Owing to their low cytotoxicity and high oral bioavailability, natural compounds or plant extracts, such as flavonoids, alkaloids, terpenoids, and coumarins, can serve as potent P-gp inhibitors [17,21]. Genistein and naringenin have been demonstrated to increase the accumulation of topotecan in K562/BCRP cells by modulating ABC transporters [22]. Curcumin has been reported to reduce the expression level of P-gp for enhancing the cytotoxicity of paclitaxel and adriamycin in SKOV3(TR) and K562/A02 cells [23,24]. Furthermore, glaucine has been shown to not only inhibit ABC transporters but also reduce the expression levels of MDR1 and MRP1 [25]. In addition to affecting the activity and expression of transporters, phytochemicals, such as glaucine, nobiletin, and antofine, can resensitize cancer cells to chemotherapeutic drugs through synergism [3].

Pinostrobin induces cancer cell apoptosis through the reactive oxygen species (ROS)–mediated extrinsic and intrinsic signaling pathways and ROS-mediated mitochondrial damage [17]. By contrast, tectochrysin has been demonstrated to induce apoptosis in prostate cancer cells by affecting the TNF-α-related apoptosis-inducing ligand and phosphatidylinositol 3-kinase/protein kinase B signaling pathways, suppressing NF-κB activity, and increasing the expression levels of death receptors in colon cancer cells [8,18]. Tectochrysin has been identified to be a potent inhibitor of ABCG2 [11]. However, its inhibitory effects on P-gp remain unclear. In the present study, we demonstrated that pinostrobin and tectochrysin affected the proliferation of drug-resistant cancer cells; nonetheless, the combination of pinostrobin or tectochrysin with chemotherapeutic drugs exhibited substantial cytotoxicity. Pinostrobin and tectochrysin enhanced the cytotoxicity of chemotherapeutic drugs by inhibiting the efflux of chemotherapeutic drugs and exerted synergistic effects on MDR cancer cells to resensitize them to chemotherapeutic drugs.

In terms of P-gp binding sites, the H-site preferentially interacts with Hoechst 33258, Hoechst 33342, colchicine, and quercetin, whereas the R-site preferentially binds daunorubicin, rhodamine 123, doxorubicin, and other anthracyclines. Furthermore, compounds that block substrate efflux may bind to the M-site on P-gp, which is a modulator site. The P-gp substrates rhodamine 123 and doxorubicin may recognize and bind to the R-site, whereas rhodamine 123 binds to the M-site [26]. In our kinetic analysis, pinostrobin and tectochrysin noncompetitively inhibited drug (rhodamine 123 and doxorubicin) efflux through P-gp. This finding implies that pinostrobin and tectochrysin bind to the H-site or other unknown binding sites on P-gp. Through docking analysis, we observed that the two test compounds had similar binding residues on P-gp, which differed from those of verapamil. The level of the lowest binding energy was higher for verapamil than for pinostrobin and tectochrysin. The torsdof parameter, which indicates free rotatable bonds, revealed that verapamil has several rotatable bonds. This explains the difficulty in predicting optimal docking of verapamil with P-gp [27].

In addition to drug-binding sites, P-gp has two ATP-binding sites. Compared with basal activity, ATPase activity was inhibited in the presence of pinostrobin and tectochrysin. Pinostrobin and a high concentration of tectochrysin inhibited the verapamil-stimulated ATPase activity of P-gp and the concentrations of pinostrobin and tectochrysin were found to be inversely correlated with ATPase activity. This finding suggests that the affinities of pinostrobin and tectochrysin for ATPase may be stronger than that of verapamil. Regarding the conformation of P-gp, vinblastine—a substrate of P-gp—markedly increased fluorescence and altered P-gp conformation. By contrast, pinostrobin and tectochrysin reduced fluorescence and slightly modified the P-gp conformation. The effects of the test compounds were weaker than that of vinblastine. Therefore, pinostrobin and tectochrysin may not be the substrates of P-gp. This is advantageous because had the compounds been P-gp substrates, higher concentrations of the compounds would have been required for the effective inhibition of P-gp-mediated drug efflux.

Our study has several strengths. For example, we used a cell line that stably expressed human P-gp (*ABCB1*/Flp-In^TM^-293) to elucidate the mechanisms underlying the effects of test compounds; this avoided interference from other transporters. Furthermore, to mimic the tumor environment in clinical conditions, we used an MDR cancer cell line. Compared to previous three generations of inhibitors, pinostrobin and tectochrysin, as natural compounds, have lower cytotoxicity and better oral bioavailability. With low cytotoxic profiles, these natural products may serve as daily supplements and may provide better disease control via increasing efficacy of chemotherapeutic agents. In addition, they may consider as leads for future drug designs. Previously, Mohana et al. [16] have demonstrated that the results of pinostrobin docking to P-gp and QSAR prediction. They did not mention any results about tectochrysin and conduct any in vitro experiments related to these compounds. In addition, Ahmed-Belkacem, A et al. [15] have evidenced that tectochrysin is a potent inhibitor of ABCG2-mediated drug efflux but have not investigated the effect on P-gp activity in depth. To the best of our knowledge, the present study is the first to investigate the inhibitory effects of pinostrobin and tectochrysin on P-gp and the underlying molecular mechanisms.

The major limitation of the present study is a lack of in vivo evidence. In a relevant study [12], the anticancer effect of tectochrysin was investigated in an animal model; however, more in vivo studies are needed to validate the efficacy of the test compounds for MDR reversal. The overexpression of P-gp might play a crucial role in clinical drug resistance in a few cases, not in most drug-resistant patients [18,28].

In the present study, we investigated the MDR reversal potential and P-gp inhibitory effects of pinostrobin and tectochrysin. Our findings revealed the detailed mechanisms underlying the inhibitory effects of the aforementioned compounds on ATP consumption and human P-gp conformation. Pinostrobin and tectochrysin may be promising P-gp modulators. This study may serve as a reference for future studies aimed at developing treatment strategies for patients with MDR cancer.

## 4. Materials and Methods

### 4.1. Chemical Compounds and Reagents

Pinostrobin, tectochrysin, dimethyl sulfoxide (DMSO), acetic acid, ethanol (analytical and absolute grades), β-mercaptoethanol (β-ME), sulforhodamine B (SRB), Tris base, rhodamine 123, paclitaxel, vincristine, verapamil, calcein-AM, and trichloroacetic acid (TCA) were obtained from Sigma-Aldrich Co. (St Louis, MO, USA). Doxorubicin was purchased from United States Biological (Woburn, Massachusetts, USA). Zeocin was obtained from InvivoGen (San Diego, CA, USA). Roswell Park Memorial Institute (RPMI) 1640 medium and Dulbecco’s Modified Eagle Medium (DMEM) were purchased from Thermo Fisher Scientific Inc. (Waltham, MA, USA). Phosphate buffered saline (PBS), hygromycin B, fetal bovine serum (FBS), and Trypsin-ethylenediamine tetra-acetic acid were obtained from Invitrogen (Carlsbad, CA, USA).

### 4.2. Cell Culture

Drug-sensitive HeLa S3 and KB cancer cell lines were from Bioresource Collection and Research Center (Hsinchu, Taiwan). The KBvin cell line was a generous gift from Dr. Kuo-Hsiung Lee (University of North Carolina, Chapel Hill, NC, USA). The parental cells Flp-In^TM^-293 were incubated with 100 μg/mL zeocin. ABCB1-expressing cells (*ABCB1*/Flp-In^TM^-293) with the stable expression of human P-gp were cultured with 100 μg/mL hygromycin B. The Flp-In^TM^-293 and *ABCB1*/Flp-In^TM^-293 cell lines were established as described previously [29]. A total of five cell lines were incubated in DMEM or RPMI 1640 with 10% FBS and incubated at 37 °C under 5% CO_2_.

### 4.3. Cell Viability Assay and CI Analysis

The cells were treated with different concentrations of chemotherapeutic drugs, pinostrobin, tectochrysin, or their combination (pinostrobin or tectochrysin + chemotherapeutic drug) for 72 h. Then, 50% TCA was used to fix the living cells for 30 min. Subsequently, 0.04% SRB was used to stain the fixed cells for 30 min. Tris base (10 mM) was used to dissolve the attached SRB. Absorbance was recorded at 515 nm using a BioTek Synergy HT Multi-Mode Microplate Reader (Agilent, Santa Clara, CA, USA). Using CompuSyn software, CI values were determined on the basis of the IC_50_ values of the test compounds and chemotherapeutic drugs. CI values of <1, 1, and >1, respectively, indicate synergism, additive effect, and antagonism in drug combination [30].

### 4.4. Cell Proliferation Assay

The effects of pinostrobin and tectochrysin on cell proliferation were evaluated using the CFSE Cell Labeling Kit (ab113853; Abcam, Cambridge, UK). The cells were harvested and suspended in 2 mL PBS. CFSE stock solution (10 mM; 2 μL) was added to the cell suspension and incubated for 10 min at 37 °C. Next, the stained cells were washed with medium to remove the excess dye and were seeded in 6-well plates, which were subjected to pinostrobin or tectochrysin treatment for 72 h. Subsequently, the cells were harvested and resuspended in PBS. Fluorescence-activated cell sorting (FACS) analysis (BD FACSCanto^TM^ II System; fluorescein isothiocyanate [FITC] channel) was performed to measure fluorescence levels. Relative division index was calculated by dividing the geometric mean of 0-h CFSE fluorescence with the CFSE fluorescence of different time points [31].

### 4.5. Calcein-AM Uptake assay

A calcein-AM uptake assay was performed to assess the effects of pinostrobin and tectochrysin on the efflux function of human P-gp. The density of the cells in 96-well plates was 1 × 10^5^ cells/well. After incubation for 24 h, the cells were washed and pretreated with pinostrobin, tectochrysin, or verapamil for 30 min. Then, they were washed and treated with calcein-AM for 30 min at 37 °C. The excitation and emission wavelengths were 485 and 528 nm, respectively. Using BioTek Synergy HT Multi-Mode Microplate Reader, fluorescence levels due to intracellular calcein were measured at 37 °C every 3 min for 30 min.

### 4.6. Rhodamine 123 and Doxorubicin Efflux Assays

The densities of *ABCB1*/Flp-In^TM^-293 and Flp-In^TM^-293 cells in 96-well plates were 1 × 10^5^ and 1.4 × 10^5^ cells/well, respectively. After 24 h of incubation, the cells were treated with pinostrobin or tectochrysin for 30 min. Then, they were incubated at 37 °C with rhodamine 123 for 30 min or doxorubicin for 1.5 h. Next, warm PBS was used to wash the cells, which were incubated for 10 and 25 min, respectively. The supernatant was added to 96-well black plates. A SpectraMax iD3 Multi-Mode Microplate Reader (Molecular Devices, San Jose, CA, USA) was used to measure fluorescence levels without light exposure. The excitation and emission wavelengths for rhodamine 123 were 485 and 528 nm, respectively; the corresponding wavelengths for doxorubicin were 485 and 590 nm, respectively.

### 4.7. MDR1 Shift Assay

Conformational changes in human P-gp were investigated by performing an MDR1 shift assay (MDR1 Shift Assay Kit; EMD Millipore Corp., Billerica, MA, USA). UIC2 antibodies can serve as conformation-sensitive antibodies against human MDR1. Upon the attachment of a substrate to P-gp, UIC2 binds to the open conformation of P-gp. For the assay, the cells were first treated with vinblastine (positive control), DMSO (solvent control), pinostrobin, or tectochrysin for 30 min. Then, they were incubated with the working solution of UIC2 or IgG2a (negative control antibody) for 15 min at 37 °C. After centrifugation at 200× *g* for 10 min, Alexa Fluor^®^ 488 (MDR1 Shift Assay Kit; EMD Millipore Corp., Billerica, MA, USA)—a secondary antibody of UIC2—was incubated with the cells for 15 min at 4 °C. FACS analysis (BD FACSCanto^TM^ II System) (BD, Franklin Lakes, NJ, USA) was performed to measure fluorescence levels.

### 4.8. P-gp ATPase Activity Assay

A Pgp-Glo^TM^ assay (Promega, Madison, WI, USA) was performed to assess the effects of pinostrobin or tectochrysin on the activity of P-gp ATPase. A series of test compound concentrations was added into 96-well white plates, followed by the addition of recombinant human P-gp membranes; the mixture was incubated for 5 min at 37 °C. Verapamil was used as a positive control. Then, the cells were incubated with MgATP for 80 min at 37 °C to induce P-gp ATPase activity. To stop the reaction, the mixture was incubated with ATPase Detection Reagent for 20 min at 26 °C. SpectraMax iD3 Multi-Mode Microplate Reader was used to detect luminescence. Changes in luminescence (ΔRLU) were recorded.

### 4.9. Cell Cycle Analysis

Cancer cells were incubated with serum-free medium in 6-well plates for 24 h at 37 °C. Then, the cells were incubated with chemotherapeutic drugs or their combinations with different concentrations of test compounds for 48 h at 37 °C. After harvesting and washing (cold PBS), the cells were incubated with 70% ethanol for at least 24 h for cellular fixation. After fixation, the cells were washed again with PBS and resuspended in staining buffer (FBS). Propidium iodide (PI)/RNase Staining Buffer (500 μL; BD Pharmingen^TM^, San Diego, CA, USA) was added to the cells, and the mixture was incubated for 15 min at room temperature under dark conditions. FACS analysis (BD FACSCanto^TM^ II System) was performed to analyze the harvested cells. The excitation and emission wavelengths for PI were 488 and 575 nm, respectively. ModFit LT software is a flow cytometry analysis software. This software was used for DNA analysis to quantify cell cycle. The raw data were imported after open the software. First, the gate parameter and region would be defined and enabled gate 1 to get the right peak distribution. Then, properties about debris, aggregates, and apoptosis were edited for manual analysis. Finally, the ranges of different cell phases would be regulated and fitted the selected peaks. The percent of each phase in cell cycle would be produced and used to be calculated.

### 4.10. Apoptosis Assay

The pattern of cell death was investigated using Annexin V-FITC/PI Apoptosis Detection Kit (Elabscience^®^, Houston, TX, USA). The cells in 6-well plates were treated with chemotherapeutic drugs with or without the test compounds at 37 °C for 48 h. Subsequently, they were harvested and washed with cold PBS and resuspended in Annexin V Binding Buffer (1×; 500 mL). Subsequently, 5 μL of Annexin V-FITC or PI solution was added to 100 μL of the aforementioned suspension and incubated for 15 min at room temperature under dark conditions. Then, 400 μL 1× Binding Buffer was added to each mixture, which was then subjected to FACS analysis (BD FACSCanto^TM^ System). The excitation wavelength was 488 nm; the emission wavelengths for FITC and PI were 530 and 575 nm, respectively.

### 4.11. Western Blotting

Protein expression was assessed through Western blotting. KB and KBvin cells were plated in 6-well plates and treated with different concentrations of the test compounds for 48 h. The harvested cells were lysed in radioimmunoprecipitation assay buffer for 5 min at 4 °C. The suspensions were heated at 70 °C for 10 min and separated through sodium dodecyl sulfate–polyacrylamide gel electrophoresis. Subsequently, the separated bands on gels were transferred onto nitrocellulose membranes. The membranes were blocked with 5% nonfat milk for 1 h and incubated with antibodies for β-actin and P-gp at 4 °C overnight. Then, the blots were incubated with secondary antibodies for 1 h at 4 °C, followed by incubation with electrochemiluminescence reagent for 5 min at room temperature. Chemiluminescence levels were measured using Cytiva ImageQuant™ 800 (Cytiva, Marlborough, MA, USA). Data were analyzed using Image Lab (version 6.0).

### 4.12. Molecular Docking

AutoDock (version 4.2.6) was used to simulate molecular docking, calculate the lowest binding energy, and identify the best binding site. The crystal structure of human P-gp (Protein Data Bank [PDB] ID: 7A65) [32] was downloaded from the Research Collaboratory for Structural Bioinformatics PDB. Ligand docking at the binding sites of P-gp was simulated using the standard procedure. A grid box (size, 70 Å × 70 Å × 70 Å) was centered at the coordinates 164.072 (*x*-axis), 157.954 (*y*-axis), and 159.047 (*z*-axis) of the PDB structure; 0.500 spacing was ensured. The population size was 300, and the number of genetic algorithm run was 50. LigPlus (version 2.2.4) was used to generate two-dimensional plots. The data were used to perform energy-based measurements and visualize and classify drug interactions.

### 4.13. Statistical Analysis

Student’s *t*-tests were performed to determine statistical differences. Statistical significance was set at *p* < 0.05.

## Figures and Tables

**Table 1:** Kinetic parameters of P-gp-mediated effluxes of rhodamine 123 and doxorubicin in ABCB1/Flp-In™-293 cells treated with pinostrobin.

	**Nonlinear Kinetic Parameters**	
	**V_m_ (pmol/mg Protein/10 min)**	**K_m_ (μM)**
Nonlinear regression		
Rhodamine 123 only	16.27 ± 0.43	12.86 ± 0.78
+50 μM Pinostrobin	13.77 ± 0.29 *	12.22 ± 0.50
+100 μM Pinostrobin	11.45 ± 0.15 *	12.42 ± 0.24
	**V_m_ (pmol/mg Protein/25 min)**	**K_m_ (μM)**
Nonlinear regression		
Doxorubicin only	1.80 ± 0.06	0.06 ± 0.004
+50 μM Pinostrobin	1.63 ± 0.01 *	0.06 ± 0.002
+100 μM Pinostrobin	1.32 ± 0.01 *	0.06 ± 0.002

V_m_ is the maximal efflux rate of rhodamine 123 or doxorubicin. K_m_ represents the Michaelis–Menten constant. * indicates comparison with nontreatment (rhodamine 123 or doxorubicin only); *p* < 0.05.

**Table 2:** Kinetic parameters of human P-gp-mediated effluxes of rhodamine 123 and doxorubicin in ABCB1/Flp-In™-293 cells treated with tectochrysin.

	**Nonlinear Kinetic Parameters**	
	**V_m_ (pmol/mg Protein/10 min)**	**K_m_ (μM)**
Nonlinear regression		
Rhodamine 123 only	14.8 ± 0.57	17.33 ± 0.70
+50 μM Tectochrysin	10.64 ± 0.15 *	16.45 ± 0.47
+100 μM Tectochrysin	7.72 ± 0.11 *	16.04 ± 0.53
	**V_m_ (pmol/mg Protein/25 min)**	**K_m_ (μM)**
Nonlinear regression		
Doxorubicin only	3.74 ± 0.04	0.14 ± 0.002
+50 μM Tectochrysin	3.12 ± 0.04 *	0.14 ± 0.004
+100 μM Tectochrysin	2.85 ± 0.004 *	0.14 ± 0.001

V_m_ is the maximal efflux rate of rhodamine 123 or doxorubicin. K_m_ represents the Michaelis–Menten constant. * indicates comparison with nontreatment (rhodamine 123 or doxorubicin only); *p* < 0.05.

**Table 3:** IC_50_ values (72 h) of pinostrobin and tectochrysin in four cell lines.

Cell Lines	IC_50_ (μM) ± SE	
	Pinostrobin	Tectochrysin
Flp-In™-293	666.64 ± 7.01	281.58 ± 1.84
*ABCB1*/Flp-In™-293	510.53 ± 4.85	242.17 ± 2.67
HeLa S3	530.05 ± 12.37	207.37 ± 2.88
KBvin	623.59 ± 68.65	229.87 ± 2.05

SE, standard error.

**Table 4:** IC_50_ values (72 h) of the combination of pinostrobin or tectochrysin with three different chemotherapeutic drugs in HeLa S3 and KBvin cells.

Treatment	HeLa S3 (Drug Sensitive)		KBvin (Resistant)	
	IC_50_ (nM) ± SE	RF	IC_50_ (nM) ± SE	RF
Vincristine	6.30 ± 0.07		2860.66 ± 24.65	
+50 μM Pinostrobin	2.78 ± 0.03 *	2.27	332.24 ± 5.60 *	8.61
+100 μM Pinostrobin	0.95 ± 0.01 *	6.63	252.03 ± 4.42 *	11.35
+50 μM Tectochrysin	4.01 ± 0.03 *	1.57	382.19 ± 17.63 *	7.48
+100 μM Tectochrysin	2.38 ± 0.07 *	2.65	149.66 ± 20.86 *	19.11
Paclitaxel	10.36 ± 0.30		1178.73 ± 28.45	
+50 μM Pinostrobin	7.11 ± 0.04 *	1.46	217.94 ± 7.68 *	5.41
+100 μM Pinostrobin	5.90 ± 0.10 *	1.76	73.84 ± 1.16 *	15.96
+50 μM Tectochrysin	8.74 ± 0.24 *	1.19	202.09 ± 15.78 *	5.83
+100 μM Tectochrysin	5.34 ± 0.09	1.94	60.23 ± 0.13 *	19.57
Docetaxel	5.29 ± 0.07		267.33 ± 13.27	
+50 μM Pinostrobin	2.17 ± 0.10 *	2.44	18.90 ± 0.63 *	14.14
+100 μM Pinostrobin	0.64 ± 0.02 *	8.27	113.97 ± 0.69 *	19.13
+50 μM Tectochrysin	3.29 ± 0.14	1.61	8.85 ± 0.41 *	30.20
+100 μM Tectochrysin	0.61 ± 0.02 *	8.64	3.25 ± 0.13 *	82.26

RF, reversal fold; SE, standard error. RF = IC_50_ values of chemotherapeutic drugs divided by those of the combination of chemotherapeutic drugs with either pinostrobin or tectochrysin. * indicates comparison with noncombination (chemotherapeutic drugs only); *p* < 0.05.

**Table 5:** IC_50_ values (72 h) of the combination of pinostrobin or tectochrysin with three different chemotherapeutic drugs in Flp-In™-293 and ABCB1 overexpressing Flp-In™-293 cells.

Treatment	Flp-In™-293 Cell (Parental)		*ABCB1*/Flp-In™-293 Cell (Resistant)	
	IC_50_ (nM) ± SE	RF	IC_50_ (nM) ± SE	RF
Vincristine	7.62 ± 0.11		855.97 ± 11.91	
+50 μM Pinostrobin	1.09 ± 0.05 *	6.99	98.93 ± 1.40 *	8.65
+100 μM Pinostrobin	0.78 ± 0.02 *	9.79	60.21 ± 0.98 *	14.22
+50 μM Tectochrysin	3.95 ± 0.03 *	1.93	91.71 ± 1.70 *	9.33
+100 μM Tectochrysin	3.01 ± 0.12 *	2.53	62.94 ± 0.67 *	13.60
Paclitaxel	12.98 ± 1.72		850.14 ± 19.34	
+50 μM Pinostrobin	6.87 ± 0.12	1.89	231.67 ± 10.05 *	3.67
+100 μM Pinostrobin	5.69 ± 0.07	2.28	41.70 ± 1.76 *	20.39
+50 μM Tectochrysin	7.86 ± 0.14	1.65	90.79 ± 1.91 *	9.36
+100 μM Tectochrysin	7.32 ± 0.17	1.77	68.59 ± 1.57 *	12.39
Docetaxel	6.92 ± 0.12		140.86 ± 11.60	
+50 μM Pinostrobin	5.15 ± 0.17 *	1.34	22.47 ± 1.00 *	6.27
+100 μM Pinostrobin	1.40 ± 0.14 *	4.95	3.96 ± 0.24 *	35.61
+50 μM Tectochrysin	5.61 ± 0.34 *	1.23	6.83 ± 0.11 *	20.64
+100 μM Tectochrysin	3.39 ± 0.30 *	2.04	5.35 ± 0.08 *	26.32

RF, reversal fold; SE, standard error. RF = IC_50_ values of chemotherapeutic drugs divided by those of the combination of chemotherapeutic drugs with either pinostrobin or tectochrysin. * indicates comparison with noncombination (chemotherapeutic drugs only); *p* < 0.05.

**Table 6:** Combination index values of the combinations of pinostrobin with vincristine, paclitaxel, and docetaxel in MDR KBvin cells.

Pinostrobin	[Vincristine]	Fraction Affected (Fa)	Combination Index (CI)	Drug Interaction Description
50 μM	1000 nM	0.098	0.132	Strong synergism
	100 nM	0.630	0.228	Strong synergism
100 μM	1000 nM	0.049	0.092	Very Strong Synergism
	100 nM	0.599	0.355	Synergism
Pinostrobin	[Paclitaxel]	Fraction affected (Fa)	Combination index (CI)	Drug interaction description
50 μM	100 nM	0.574	0.217	Strong synergism
	10 nM	0.740	0.336	Synergism
100 μM	100 nM	0.432	0181	Strong synergism
	10 nM	0.51	0.405	Synergism
Pinostrobin	[Docetaxel]	Fraction affected (Fa)	Combination index (CI)	Drug interaction description
50 μM	100 nM	0.026	0.093	Very Strong Synergism
	10 nM	0.540	0.197	Strong synergism
100 μM	100 nM	0.300	0.523	Synergism
	10 nM	0.505	0.275	Strong synergism

CI < 1, synergism; CI = 1, additive effect; and CI > 1, antagonism.

**Table 7:** Combination index values of the combinations of tectochrysin with vincristine, paclitaxel, and docetaxel in MDR KBvin cells.

Tectochrysin	[Vincristine]	Fraction Affected (Fa)	Combination Index (CI)	Drug Interaction Description
50 μM	1000 nM	0.126	0.215	Strong synergism
	100 nM	0.684	0.389	Synergism
100 μM	1000 nM	0.046	0.160	Strong synergism
	100 nM	0.578	0.560	Synergism
Tectochrysin	[Paclitaxel]	Fraction affected (Fa)	Combination index (CI)	Drug interaction description
50 μM	100 nM	0.594	0.357	Synergism
	10 nM	0.895	0.839	Moderate synergism
100 μM	100 nM	0.292	0.277	Strong synergism
	10 nM	0.763	0.859	Strong synergism
Tectochrysin	[Docetaxel]	Fraction affected (Fa)	Combination index (CI)	Drug interaction description
50 μM	100 nM	0.008	0.061	Very Strong Synergism
	10 nM	0.483	0.277	Strong synergism
100 μM	100 nM	0.001	0.023	Very Strong Synergism
	10 nM	0.125	0.169	Strong synergism

CI < 1, synergism; CI = 1, additive effect; and CI > 1, antagonism.

**Table 8:** Results of the molecular docking of pinostrobin, tectochrysin, and verapamil.

	Lowest Binding Energy (kcal/mol)	Mean Binding Energy (kcal/mol)	predKi	Td	CR	Number of Runs in 1st Cluster	Amino Acids Involved in Interactions	Amino Acids Involved in H-Bonds
Pinostrobin	−8.26	−8.07	882.38 nM	2	11	10	Clr1302, Cys956, Gly955, Ile864, Leu860, Leu861, Met948, Phe951, Ser119, Ser952	None
Tectochrysin	−8.17	−7.98	1.03 μM	2	7	9	Ala954, Clr1302, Cys956, Gly955, Ile864, Leu860, Leu861, Met948, Phe951, Ser119, Ser952	None
Verapamil	−5.57	−3.21	82.19 μM	17	29	4	Ala987, Gln725, Gln990, Ile306, Met986, Phe728, Phe983, Tyr307, Tyr310	None

The lowest and mean binding energy levels predicted inhibition constant (predKi) values, torsdof parameter (Td) numbers, and cluster ranks (CRs) of the test compounds and verapamil. The amino acids involved in the docking interactions are also presented.

## Data Availability

Data is contained within the article and supplementary material.

## Supplementary Materials

The following supporting information can be downloaded at: https://www.mdpi.com/article/10.3390/ph16020205/s1, Figure S1: The distribution of CFSE fluorescence was analyzed by flow cytometry at 0, 24, 48 and 72 h; Figure S2: The raw data of Western blotting. Tables S1 and S2: IC_50_ values (72 h) of only and the combination of tariquidar with paclitaxel in HeLa S3 and KBvin cells.
