# Supplementary material for: Pinostrobin and Tectochrysin Conquer Multidrug-Resistant Cancer Cells via Inhibiting P-Glycoprotein ATPase
Source: Pharmaceuticals (Basel). 2023 Jan 29;16(2):205. doi: 10.3390/ph16020205 (PMC9963356; doi:10.3390/ph16020205)
Supplement: Supplementary file 1 [file pharmaceuticals-16-00205-s001.zip › pharmaceuticals-2088300-supplementary.pdf]

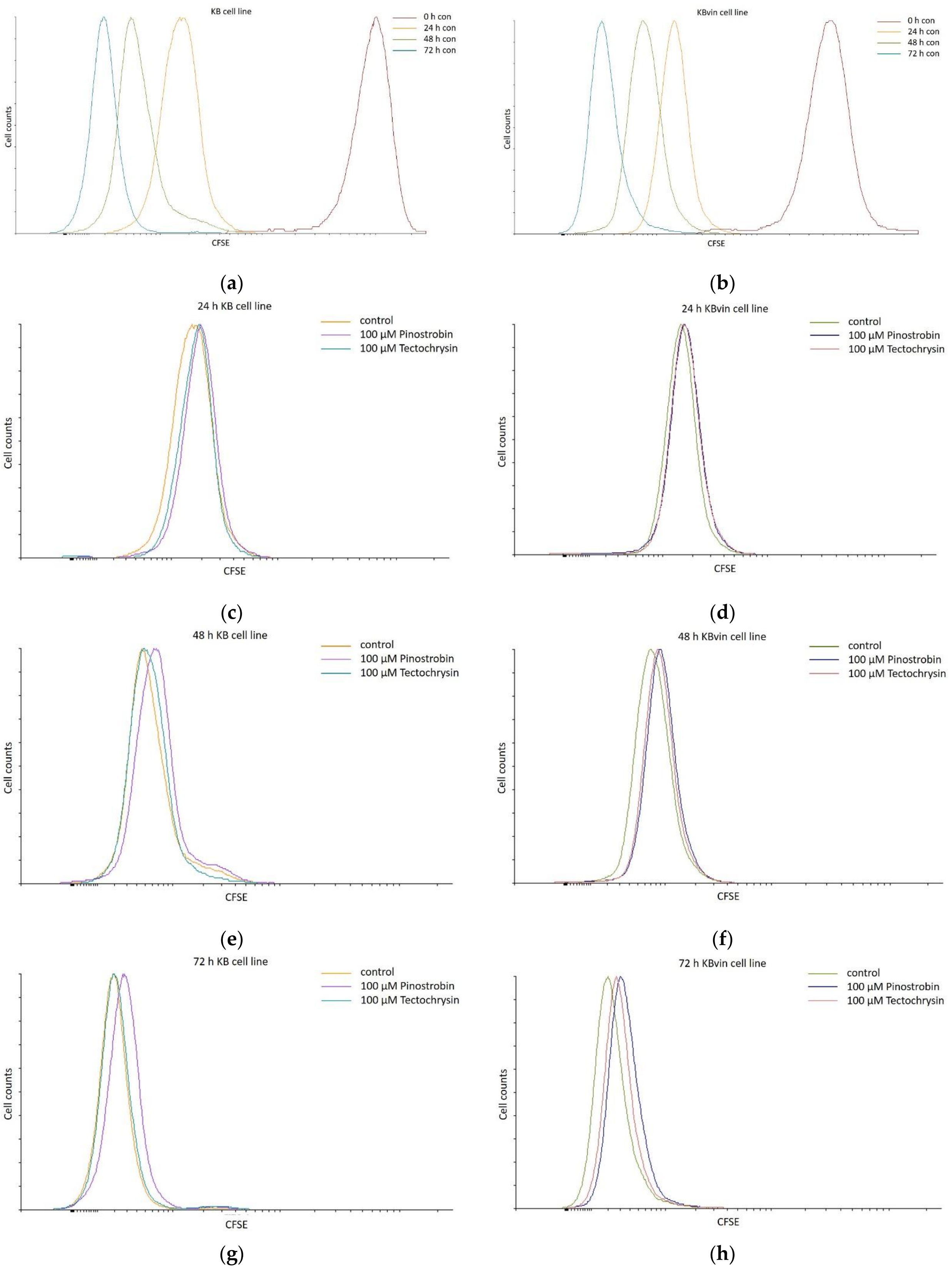

Figure S1. The distribution of CFSE fluorescence was analyzed by flow cytometry at 0, 24, 48 and 72 h. (**a**) and (**b**) were the fluorescence of KB and KBvin cells at different time points. (**c**), (**e**) and (**g**) demonstrated the fluorescence peak of KB cells after pinostrobin or tectochrysin treatment for 24 h, 48 h and 72 h, respectively. (**d**), (**f**) and (**h**) suggested the fluorescence peak of KBvin cells.

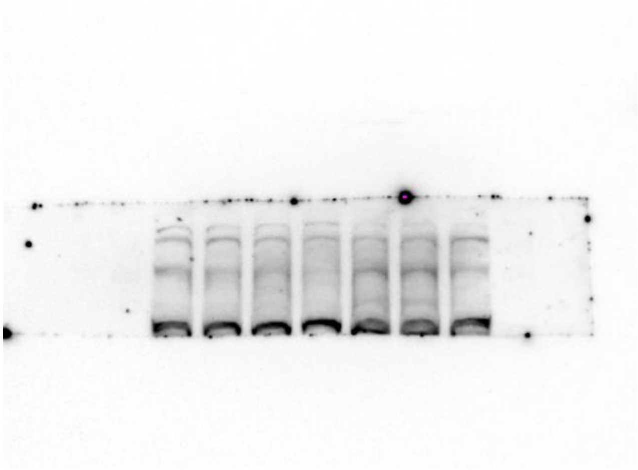

(**a**)

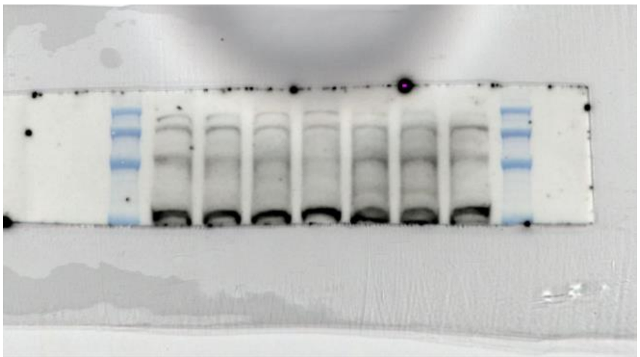

(**b**)

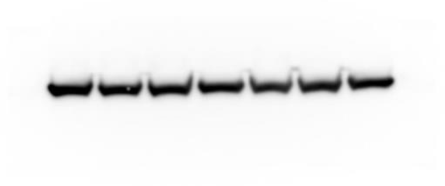

(**c**)

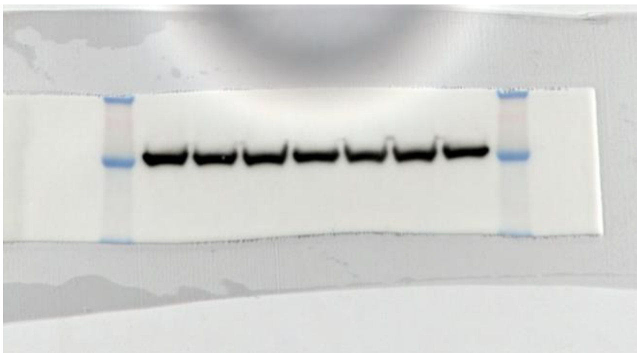

(**d**)

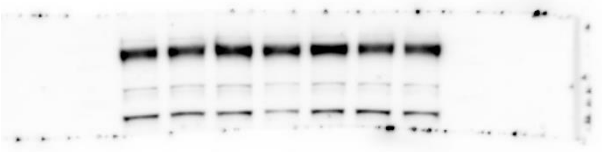

(**e**)

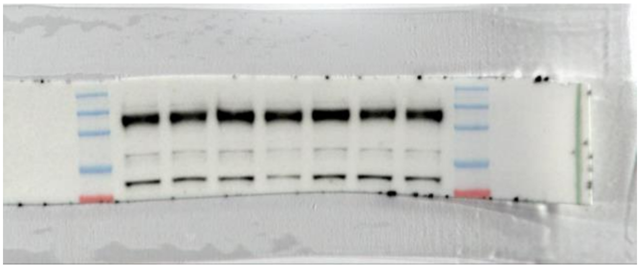

(**f**)

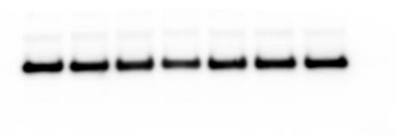

(**g**)

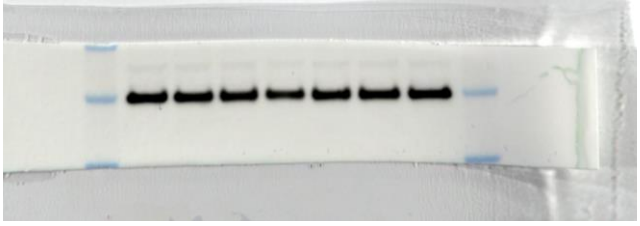

(**h**)

Figure S2. The raw data of Western blotting. (**a**), (**c**), (**e**) and (**g**) were the raw data of chemiluminescence. (**b**), (d), (f) and (h) demonstrated the data containing marker. (**a–d**) presented the bands of P-gp and beta-actin in KB cells, respectively. (e) ~ (h) indicated the bands of P-gp and beta-actin in KBvin cells.

Table S1. IC_{50} values (72 h) of tariquidar in HeLa S3 and KBvin cell lines.

**Treatment**	**HeLa S3 (drug sensitive)**	**KBvin (resistant)**
	**IC50 (μM) ± SE**	**IC50 (μM) ± SE**
Tariquidar	16.09 ±1.01	8.66 ± 0.27

SE, standard error.

Table S2. IC_{50} values (72 h) of the combination of tariquidar with paclitaxel in HeLa S3 and KBvin cells.

**Treatment**	**HeLa S3 (drug sensitive)**		**KBvin (resistant)**	
	**IC50 (nM) ± SE**	**RF**	**IC50 (nM) ± SE**	**RF**
Paclitaxel	10.36 ± 0.30		1178.73 ± 28.45	
+ 1 μM Tariquidar	1.06 ± 0.08	9.78	0.39 ± 0.17	3022.39
+ 2.5 μM Tariquidar	1.19 ± 0.25	8.71	0.24 ± 0.03	4911.38

RF, reversal fold; SE, standard error. RF = IC_{50} values of paclitaxel divided by those of the combination of paclitaxel with tariquidar.
